# miR-277 regulates the phase of circadian activity-rest rhythm in *Drosophila melanogaster*

**DOI:** 10.3389/fphys.2023.1082866

**Published:** 2023-11-28

**Authors:** Geo Anna, Maria John, Nisha N. Kannan

**Affiliations:** Chronobiology Laboratory, School of Biology, Indian Institute of Science Education and Research (IISER), Thiruvananthapuram, Kerala, India

**Keywords:** circadian, phase, amplitude, free running period, miR-277, light intensity, Clock gene

## Abstract

Circadian clocks temporally organize behaviour and physiology of organisms with a rhythmicity of about 24 h. In *Drosophila*, the circadian clock is composed of mainly four clock genes: *period* (*per*)*, timeless* (*tim*)*, Clock* (*Clk*) and *cycle* (*cyc*) which constitutes the transcription-translation feedback loop. The circadian clock is further regulated via post-transcriptional and post-translational mechanisms among which microRNAs (miRNAs) are well known post-transcriptional regulatory molecules. Here, we identified and characterized the role of miRNA-277 (miR-277) expressed in the clock neurons in regulating the circadian rhythm. Downregulation of miR-277 in the pacemaker neurons expressing circadian neuropeptide, pigment dispersing factor (PDF) advanced the phase of the morning activity peak under 12 h light: 12 h dark cycles (LD) at lower light intensities and these flies exhibited less robust rhythms compared to the controls under constant darkness. In addition, downregulation of miR-277 in the PDF expressing neurons abolished the *Clk* gene transcript oscillation under LD. Our study points to the potential role of miR-277 in fine tuning the *Clk* expression and in maintaining the phase of the circadian rhythm in *Drosophila*.

## Introduction

Organisms have evolved with biological timekeeping systems to synchronize various biological processes with the cyclic environmental changes caused by earth’s rotation. The biological clock rhythmically regulates many aspects of metabolism, physiology and behaviour in diverse organisms. The clock genes that control the circadian rhythm were first identified in *Drosophila* and the fly circadian oscillatory mechanisms have mammalian counterparts (Konopka and Benzer, 1971; Sehgal et al., 1994; Allada et al., 1998; Gekakis et al., 1998; Rutila et al., 1998; Kume et al., 1999). The −24 h period of the endogenous clock is maintained by an interlocked transcription translation feedback loop (TTFL) (Hardin et al., 1990). The two basic helix-loop-helix transcription factors; CLOCK (CLK) and CYCLE (CYC) form a heterodimer and binds to the E-box located at the promoter region of the genes *period (per)* and *timeless (tim)* in *Drosophila* (Taylor and Hardin, 2008). PER and TIM form a heterodimer and upon increased accumulation, this heterodimer enters the nucleus and binds to CLK-CYC dimer through a PER-CLK interaction (Lee et al., 1999). This inhibits the transcription of *per* and *tim* by repressing the CLK-CYC transcriptional activity (Menet et al., 2010)*.* This TTFL occurs within a span of approximately 24 h and ensures robust circadian oscillation of clock gene transcripts and their proteins.

Zeitgebers are the cyclic environmental time cues capable of entraining the circadian clock, amongst which, light is considered to be the most potent zeitgeber for many organisms (Schlichting and Helfrich-Förster, 2015). Blue light photoreceptor CRYPTOCHROME (CRY), the compound eyes, ocelli and Hofbauer-Buchner (HB) eyelets are involved in the light mediated clock entrainment in *Drosophila* (Rieger et al., 2003; Veleri et al., 2007; Yoshii et al., 2016). CRY is present in a subset of the clock neurons as well as in the photoreceptor cells and resets the phase of the clock by leading to the degradation of TIM in a light-dependent manner (Ceriani et al., 1999; Busza et al., 2004). The compound eyes, ocelli and HB eyelets express a variety of rhodopsins (RH1-7), each with distinct spectral sensitivity (Sharkey et al., 2020). The clock neurons, particularly the pigment dispersing factor (PDF)-positive ventral lateral neurons (LNvs), which are responsible for the morning peak (M peak) of activity, have dendritic connections projecting to the accessory medullae (AMe) where they communicate directly with the projections from the HB eyelets and indirectly with the compound eyes via the interneurons (Helfrich-Förster et al., 2002; Li et al., 2018). In addition, the interaction between PDF-positive small ventral lateral neurons (sLNvs) and posterior dorsal neurons (DN1ps) plays an important role in governing the morning anticipatory activity under 12 h light: 12 h dark cycles (LD). The presence of either of these neuronal subsets is sufficient to drive morning anticipation (Stoleru et al., 2004; Zhang et al., 2010). The PDF neuropeptide is critical for proper timing of locomotor activity under LD and for maintenance of rhythm under constant darkness (DD) (Renn et al., 1999). In addition to PDF-positive neurons, the dorsal lateral neurons (LNds), which regulate the evening peak (E peak) of activity also have anatomical connections to the AMe (Helfrich-Förster et al., 2007).

Though the clock neuronal circuitry, as well as the clock gene transcript/protein oscillations are well studied, additional regulatory mechanisms which confer robustness to these circadian oscillations are not well understood. The importance of microRNA (miRNA) mediated posttranscriptional regulation in circadian rhythm has been recognized in previous studies (Kadener et al., 2009). miRNAs are endogenous small (19–25 nucleotides long) noncoding RNAs that repress the translation of mRNA or degrade it by forming an imperfect hybrid at the 3′ untranslated region (3′UTR) of the mRNAs (Carthew and Sontheimer, 2009). Recent studies indicate that posttranscriptional regulation by miRNAs has a potential role in maintaining the robustness of the circadian clock as well as in the proper phasing of the rhythm (Herranz and Cohen, 2010; Ebert and Sharp, 2012; Xue and Zhang, 2018). Although several miRNAs are expressed in clock neurons (Kadener et al., 2009), only a few miRNAs among these were examined systematically in *Drosophila* to elucidate their specific roles in maintaining the circadian rhythm (Kadener et al., 2009; Luo and Sehgal, 2012; Vodala et al., 2012; Chen et al., 2014; Sun et al., 2015; Garaulet et al., 2016; Zhang et al., 2016; Chen and Rosbash, 2017; Cusumano et al., 2018; Nian et al., 2019; Niu et al., 2019; Xia et al., 2020; Nian et al., 2022).

Our present study was aimed at elucidating the role of miRNA-277 (miR-277) in governing the circadian rhythm in *Drosophila*. Downregulation of miR-277 in the PDF positive neurons advanced the phase of circadian activity-rest rhythm under LD and amplitude under DD. miR-277 expressed in PDF-positive neurons is demonstrated to be important for proper activity distribution around the morning and evening peak under lower light intensities of LD. In addition, the diurnal oscillation of *Clk* mRNA was found to be dependent on the optimal expression level of miR-277 in the fly head.

## Materials and methodology

### Fly lines and maintenance

All the stocks were maintained in a temperature and humidity-controlled incubator (Panasonic MIR-154) at 25°C and 60% ± 5% relative humidity. LD light schedule was maintained in the incubators with a light intensity of 400 lux where the lights came on at Zeitgeber Time 00 (ZT00) and went off at ZT12. The Gal4 (G4) lines used were *tubulin* G4, *tim* G4/CyO, *Clk856* G4 (Orie Shafer Lab), *Pdf* G4 (Todd C Holmes Lab). miRNA sponge (SP) line used was the UAS miR-277 Sponge (BL- 61408). This line has multiple (20X) miRNA complementary sequences inserted into specific sites on the 2nd and 3rd chromosomes by *phiC31* site-specific integration. Double scrambled sponge (Scrambled Sponge) was used as the control for the sponge line (Fulga et al., 2015). This line also has similar repeats of sequences inserted into the same sites but the sequences are not complementary to any known miRNA. miR-277 was overexpressed using UAS miR-277 (Fly ORF–F002059). The *w* ^*1118*^ (BL-5905) was crossed with G4 (driver) and UAS (responder) lines to obtain the respective controls.

### Locomotor activity rhythm recording and analysis

Freshly emerged male flies were entrained under LD for 2–3 days before carrying out the locomotor activity-rest rhythm recording. Activity recording of 2–3 day old male flies was carried out under LD at 25°C in a cooled incubator (Panasonic MIR-154) using *Drosophila* Activity Monitor (Trikinetics, Waltham, MA) with 1 min bin interval. In order to analyze the locomotor activity under LD, the activity of each fly at every 15 min was normalized to the total activity of the day, multiplied by 100 and the average of 28–32 flies for 5 days was plotted as the percentage of activity against the time of the day. The morning and evening anticipation indices were calculated using the equation: sum of activity 3 h prior to light transition/sum of activity 6 h prior to light transition (Sheeba et al., 2010). The phase of the morning peak (M peak) was determined for individual flies by identifying the time point of highest activity between ZT20–02 excluding the startle response around ZT00 (Das et al., 2016). The time window of up to ZT02 was chosen because previous studies showed that the M peak of most of the wild-type strains of *Drosophila* occurs after lights-on (Helfrich-Förster, 2000). The rhythm parameters of the clock were estimated by recording the locomotor activity under constant darkness (DD) for 10 days. Since miR-277 is reported to have effects on lifespan and ageing (Esslinger et al., 2013), we started the locomotor activity recording with 1-day-old flies, which were entrained for 2 days under LD and then shifted to DD, so that the age of the flies is 13 days by the end of the DD recording. In this way, we ensured that the effect of the microRNA on the free-running period is not influenced by its role in ageing. The free-running period was estimated using Lomb-Scargle periodogram analysis using Actimetrics Clocklab software. The amplitude of the peak corresponding to the free running period value in the periodogram was used to measure the robustness of the rhythm. Flies whose peak value occurred below the threshold of significance level were considered arrhythmic and were not included in the analysis for the estimation of FRP and amplitude of the rhythm.

### mRNA isolation and quantification

Five-day old males entrained to LD were used for mRNA isolation. Three biological replicates each with 30 flies were sampled at 6 different time points ZT02, ZT06, ZT10, ZT14, ZT18, and ZT22. 30 fly heads were homogenized by crushing in 1 mL TRIzol reagent (Life Technologies), after which 0.2 mL of chloroform (Sigma-Aldrich) was added and centrifuged at 12,000 rpm for 10 min at 4°C to separate the RNA into the top aqueous layer. The separated aqueous phase containing genetic material was pipetted out and precipitated with 100% Isopropanol (Sigma). After DNase (Invitrogen) treatment and ethanol washes, the precipitate was redissolved in DEPC-treated water (Himedia). cDNA was synthesized from the total RNA using PrimeScript 1st strand cDNA synthesis kit (Takara Bio) following the manufacturer’s instructions. qPCR was performed on Bio-Rad CFX96 Touch Real-Time PCR detection system using TB Green Premix Ex Taq II (Takara) and the housekeeping gene *rp49* was used as the reference gene. *cry*, *per*, *tim,* and *Clk*, mRNA values shown are relative to *rp49* mRNA*.* The primer sequences used are listed in Supplementary Table S1.

### TaqMan miRNA assay

The total RNA was isolated from the samples plunged at ZT02 as mentioned in the previous section. Reverse transcription was performed with High-Capacity cDNA Reverse Transcription Kit (Applied Biosystems) using miR-277 (Applied Biosystems, Assay ID-000298) specific primer following manufacturer’s instruction. *2s* rRNA (Applied Biosystems, Assay ID-001766) was used as the endogenous control. The resulting cDNA was used for qRT-PCR based quantification of the miRNA. TaqMan Universal Master Mix II, with UNG (Applied Biosystems) was used along with miR-277 or *2s* rRNA specific mix of forward primer, reverse primer and TaqMan probe, as per the manufacturer’s instruction. miRNA level was normalized to the *2s* rRNA level to obtain the ΔCt value.

### Statistical analysis

The phase of the morning peak, free running period and amplitude of DD activity levels were analyzed for normal distribution by the Shapiro-Wilk test. Depending on the result, ANOVA was performed, followed by Tukey’s *post hoc* multiple comparisons (for normally distributed data) or Kruskal Wallis test was performed, followed by Dunn’s *post hoc* test (for data that are not normally distributed) to estimate the significant differences between different genotypes. Results were considered statistically significant when *p* < 0.05. The graphs were plotted with mean and error bars representing the SEM. Plotting as well as statistical tests were performed using GraphPad Prism. All the locomotor activity experiments were conducted thrice with a sample size of 28–32 flies to confirm the results. The results of one such biological replicate are shown in the figures.

The transcript level values were determined by normalizing them with the respective *rp49*. The values plotted are the mean and SEM of data obtained from 3 biological replicates. The gene expression data obtained by qPCR were analysed for cycling by fitting the expression values at different time points to a sine wave. The cosinor analysis was performed in MATLAB to estimate the peak phase of relative mRNA expression (Nelson et al., 1979). The mRNA is considered to be cycling when the *p* < 0.05. The peak phase values obtained from individual biological replicates by cosinor analysis were further tested statistically by *t*-test to confirm if there is any significant difference in peak phase between the genotypes.

## Results

### Downregulation of miR-277 in the PDF neurons advances the phase of the morning peak

**FIGURE 1:**
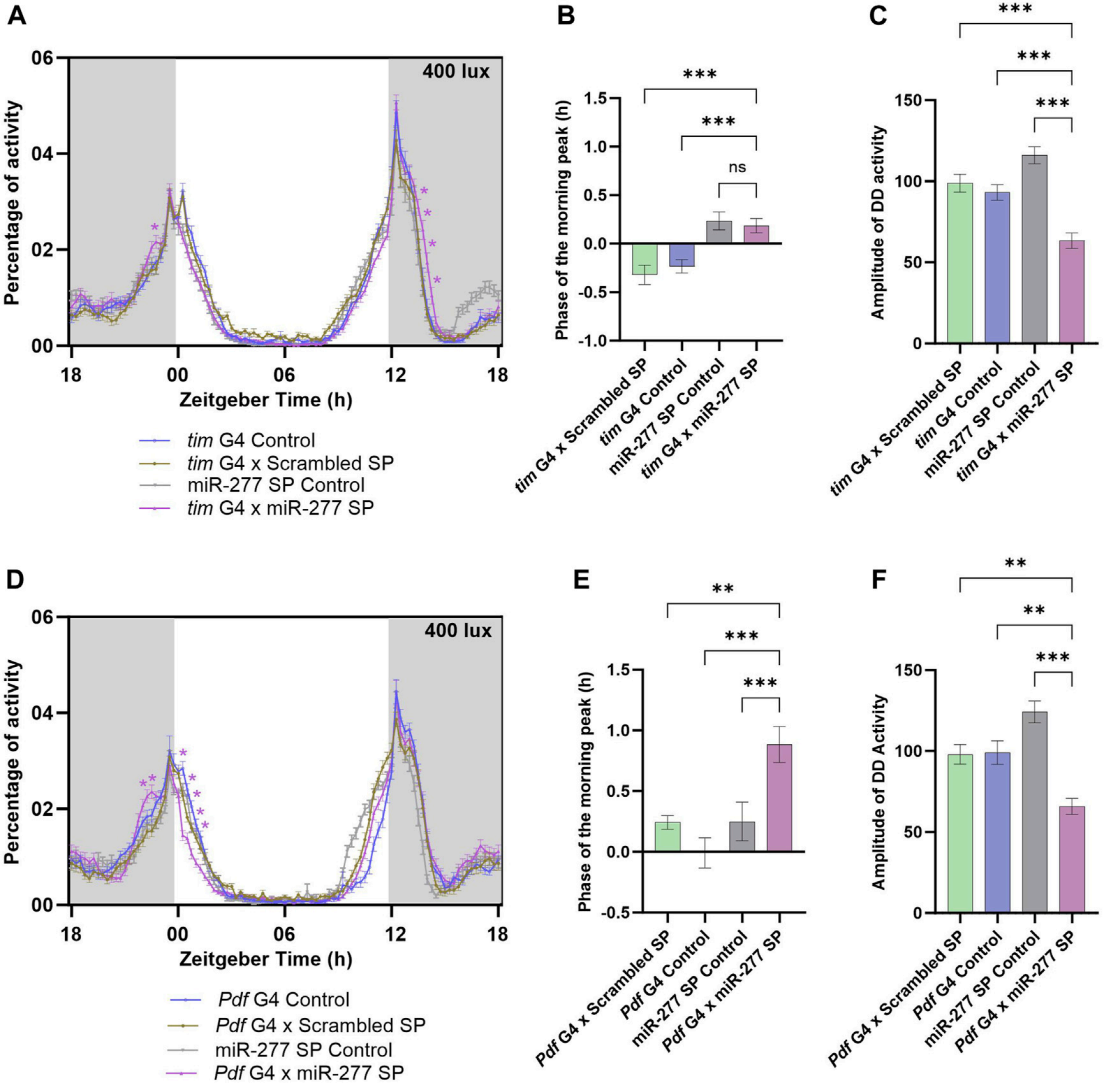
Downregulation of miR-277 in the PDF neurons advances the phase of the morning peak. **(A)** Activity rest rhythm of *tim* G4 x miR-277 SP compared to *tim* G4 control, *tim* G4 x Scrambled SP and UAS miR-277 control under LD. Locomotor activity counts were binned into 15 min intervals and percentage of activity was plotted along the *y*-axis and Zeitgeber Time in hours along the *x*-axis. Gray shaded region indicates dark phase under LD. The light intensity was maintained at 400 lux. The asterisks indicate the time points at which the experimental flies showed a significant difference compared to all the three controls. A significant increase in activity was observed at ZT 23 compared to the controls. (Two way ANOVA followed by Tukey’s Honestly Significant Difference (HSD) test. *p* < 0.05 for time point ZT23). The activity around Evening peak (E peak) also showed significant differences compared to the controls (Two way ANOVA, Tukey’s HSD test. *p* < 0.05 for time points ZT13.75- ZT14.50). **(B)** The phase of the morning peak of activity of the *tim* G4 x Scrambled SP, *tim* G4 control, UAS miR-277 control and *tim* G4 x miR-277 SP under LD. A positive value indicates phase advancement and negative value indicates phase delay. Though the phase was significantly advanced in *tim* G4 x miR-277 SP line compared to two controls, it was not significantly different from that of the UAS miR-277 SP control (Kruskal Wallis test followed by Dunn’s *post hoc* multiple comparisons. *tim* G4 x Do. Scr. Sponge vs. *tim* G4 x miR-277 Sponge *p* < 0.001, *tim* G4 Control vs. *tim* G4 x miR-277 Sponge *p* < 0.001, UAS miR-277 SP control vs. *tim* G4 x miR-277 SP *p* < 0.73). **(C)** Amplitude of the activity rest rhythm under DD was found to be decreased in *tim* G4 x miR-277 SP when compared to the controls (ANOVA, Tukey’s HSD test. *tim* G4 x Scrambled SP vs. *tim* G4 x miR-277 SP *p* < 0.001, *tim* G4 Control vs. *tim* G4 x miR-277 Sponge *p* < 0.001, UAS miR-277 SP Control vs. tim G4 x miR-277 SP *p* < 0.001). **(D)** Activity-rest rhythm under LD showing increased activity in the time points prior to lights-on in *Pdf* G4 x miR-277 SP compared to the controls (Two way ANOVA, Tukey’s HSD test. *p* < 0.05 for time points ZT22.5 and ZT22.75). **(E)** Phase of the morning peak of activity showing significant advance in *Pdf* G4 x miR-277 SP compared to the controls (Kruskal Wallis, Dunn’s *post hoc* multiple comparisons. *Pdf* G4 x Scrambled SP vs. *Pdf* G4 x miR-277 SP *p* < 0.004, *Pdf* G4 Control vs. *Pdf* G4 x miR-277 SP *p* < 0.001, UAS miR-277 SP control vs. *Pdf* G4 x miR-277 SP *p* < 0.001). **(F)** Amplitude of the rhythm under DD showing significant decrease in *Pdf* G4 x miR-277 SP compared to the controls (ANOVA, Tukey’s HSD test. *Pdf* G4 x Scrambled SP vs. *Pdf* G4 x miR-277 SP *p* < 0.002, *Pdf* G4 Control vs. *Pdf* G4 x miR-277 Sponge *p* < 0.002, UAS miR-277 SP control vs. *Pdf* G4 x miR-277 SP *p* < 0.001).

To understand the functional significance of miR-277 expressed in the clock neuron, we downregulated miR-277 in the clock neurons using *tim* G4 driver and UAS miR-277 Sponge (SP) line. The miR-277 SP line used in this study was obtained from BDSC and has been used in several previous studies (Cerro-Herreros et al., 2016; Zipper et al., 2022). These studies report that this sponge line significantly reduces the miR-277 levels. Under LD with a light intensity of 400 lux, the miR-277 downregulated flies exhibited an increased activity prior to lights-on at ZT23 (Figure 1A). This is possible when there is a phase advance in the morning peak, which was assessed by calculating the phase of the morning peak. It was found that the phase of the M peak was advanced with respect to that of the G4 control and Scrambled Sponge control (Scrambled SP); however, there was no significant advancement with respect to that of the UAS miR-277 SP control (Figure 1B). In addition, miR-277 downregulated flies exhibited an increase in activity after lights-off (ZT13.75–14.50) (Figure 1A). The locomotor activity rhythm of the flies was also recorded under DD to assess the role of miR-277 in the regulation of the free-running period (FRP) and amplitude of the circadian rhythm. Though FRP of the experimental line was not significantly different from that of the controls (Supplementary Figure S1A), the amplitude of DD activity was found to be significantly decreased in the experimental flies (Figure 1C). This suggests that miR-277 present in the clock neuronal circuit helps in maintaining robust rhythms under DD.

The PDF positive LNvs and DN1ps are the circadian neurons that regulate the morning locomotor activity behaviour in *Drosophila* (Grima et al., 2004; Stoleru et al., 2004; Zhang et al., 2010). As miR-277 downregulation in clock neurons was found to have an effect on the activity prior to the lights-on, we further restricted the downregulation of miR-277 to PDF neurons. Under LD, the morning activity was increased prior to lights-on (ZT22.5 and 22.75) in the experimental flies compared to the controls (Figure 1D). The phase of the morning peak was also found to be advanced in the experimental flies (Figure 1E). The miR-277 downregulated flies exhibited significantly reduced amplitude of activity rest rhythm under constant darkness (Figure 1F). This reduction in amplitude was observed only when DD recording was conducted using younger (3 day old) flies and not when 9 day old flies were used (Data not shown). However, the FRP of the rhythm was not affected in these flies (Supplementary Figure S1B). Thus, we conclude that miR-277 present in the PDF neurons functions in the proper phasing of the morning peak of activity and maintains robustness of the circadian rhythm under DD in *Drosophila*.

### Overexpression of miR-277 in the clock neurons advances the morning peak phase

**FIGURE 2:**
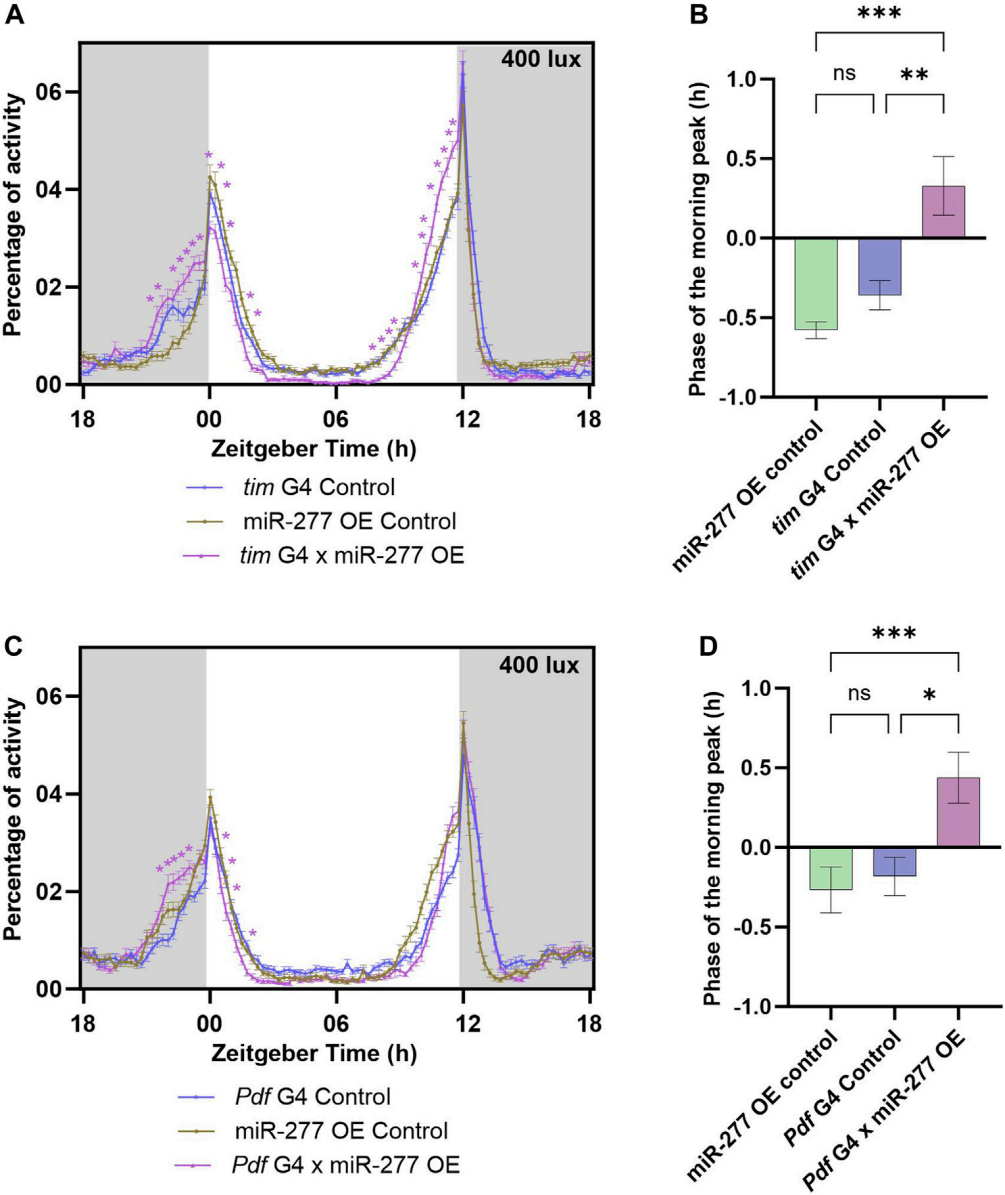
Overexpression (OE) of miR-277 in the clock neurons affects the phasing of the morning peak of activity-rest rhythm. **(A)** Activity rest rhythm of *tim* G4 x miR-277 OE flies compared to *tim* G4 control and miR-277 OE control. miR-277 overexpressed flies exhibited increased activity prior to lights on (Two way ANOVA, Tukey’s HSD test. *p* < 0.05 for time points ZT22.75- ZT23.75). The time points around the E peak also showed significant difference in activity (Two way ANOVA, Tukey’s HSD test. *p* < 0.05 for time points ZT10.25–12). The asterisks indicate the time points at which the experimental flies showed a significant difference compared to both the G4 and UAS control. **(B)** The phase of morning peak of the three genotypes under LD. The miR-277 overexpressed flies showed a significant advance in the phase of morning peak compared to the *tim* G4 and miR-277 OE controls (Kruskal Wallis test, Dunn’s *post hoc* multiple comparisons. miR-277 OE control vs. *tim* G4 x miR-277 OE *p* < 0.001, *tim* G4 Control vs. *tim* G4 x miR-277 OE *p* < 0.01). **(C)** Activity-rest rhythm of *Pdf* G4 x miR-277 OE flies compared to *Pdf* G4 control and miR-277 OE control flies. *Pdf* G4 x miR-277 OE flies have increased activity prior to lights-on (Two way ANOVA, Tukey’s HSD test. *p* < 0.05 for time points ZT22.25–23.25). **(D)** Phase of the morning peak of activity showing a significant advance in the *Pdf* G4 x miR-277 OE flies compared to *Pdf* G4 control and miR-277 OE control (Kruskal Wallis test, Dunn’s *post hoc* multiple comparisons. miR-277 OE control vs. *Pdf* G4 x miR-277 OE *p* < 0.001, *Pdf* G4 Control vs. *Pdf* G4 x miR-277 OE *p* < 0.01).

The efficiency of the overexpression line used in this study was tested by TaqMan miRNA assay. The miR-277 overexpression line showed a significant increase in miR-277 level when compared to the *tubulin* G4 and miR-277 OE Controls (Supplementary Figure S1C). The effect of overexpression of miR-277 in the clock neurons was studied by using *tim* G4 to drive the expression of miR-277. Overexpression of miR-277 using *tim* G4 resulted in an increased activity prior to lights-on (ZT22.75–23.75) under LD (Figure 2A). The phase of the morning peak was found to be advanced upon phase calculation (Figure 2B). These flies also exhibited an increase in the activity prior to lights-off (ZT10.25–12) (Figure 2A). The FRP and amplitude of these flies were estimated under DD. It was found that the FRP of the experimental flies was significantly increased compared to UAS control and significantly decreased compared to the G4 control, hence no conclusion could be drawn regarding the effect of overexpression in the clock neurons (Supplementary Figure S1D). The amplitude of the rhythm was also not different from that of the controls (Supplementary Figure S1E). The overexpression of miR-277 was then carried out in the PDF positive neurons using *Pdf* G4. This too resulted in an increased activity prior to lights-on (ZT22.25–23.25) (Figure 2C) and the phase of the morning peak was also found to be significantly advanced in the experimental flies compared to both the controls (Figure 2D). However, overexpression of miR-277 in PDF positive clock neurons did not exert any significant influence on the activity prior to or after lights-off (Figure 2C). The FRP of the miR-277 overexpressed flies was found to be significantly increased and decreased compared to the UAS control and *Pdf* G4 control respectively, which prevented us from drawing any meaningful conclusion (Supplementary Figure S1F). We did not observe any significant difference in the amplitude of activity-rest rhythm under DD compared to the controls (Supplementary Figure S1G). These results suggest that miR-277 expressed in the clock neurons is involved in the phasing of the morning peak.

### Role of miR-277 in phasing of the morning peak under different light intensities

**FIGURE 3:**
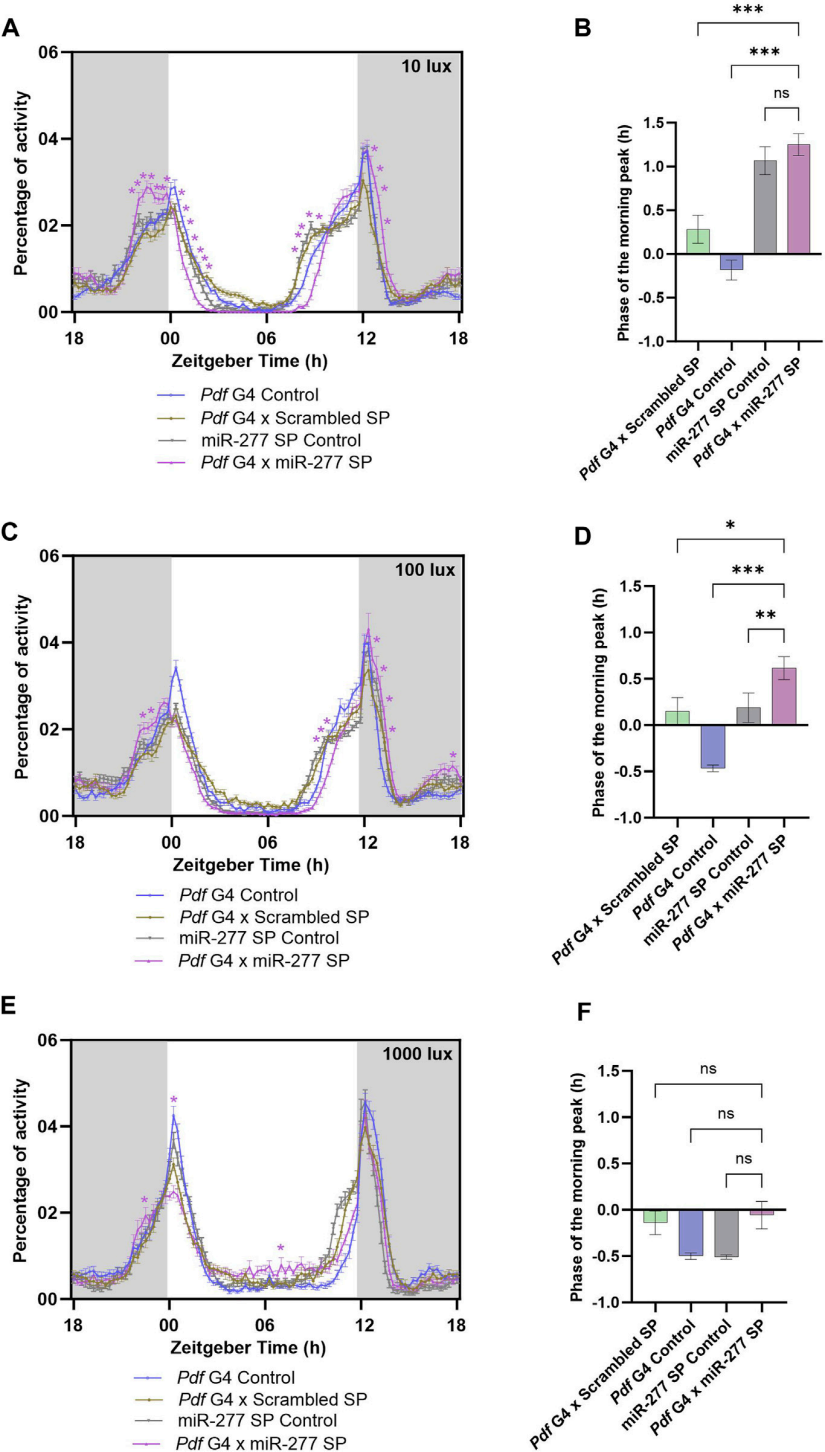
**(A, C, E)** Activity-rest rhythm of *Pdf* G4 control, *Pdf* G4 x Scrambled SP, UAS miR-277 SP control and *Pdf* G4 x miR-277 SP flies under LD with 10 lux, 100 lux and 1000 lux intensities respectively. *Pdf* G4 x miR-277 SP flies exhibited a significantly increased activity prior to lights-on compared to *Pdf* G4 control and *Pdf* G4 x Scrambled SP control under 10 and 100 lux of light intensities (Two way ANOVA, Tukey’s HSD test. *p* < 0.05 for time points ZT22.25-ZT23.50 under 10 lux. *p* < 0.05 for time points ZT22.75 and ZT23 under 100 lux). The activity around the E peak also was significantly altered (Two way ANOVA, Tukey’s HSD test. *p* < 0.05 for time points ZT08.50–09.50 and ZT12.75–13.50under 10 lux, *p* < 0.05 for time points ZT09.75–10.25 and ZT13.25–13.75 under 100 lux). Under 1000 lux light intensity, *Pdf* G4 x miR-277 SP flies showed a significant increase in the locomotor activity compared to the controls at ZT 22.75 but a decreased activity at ZT00 and ZT00.25 (Two way ANOVA, Tukey’s HSD test. *p* < 0.05 for time points ZT22.75, ZT00 and ZT00.25) **(B, D)** Under 10 lux, the phase of the morning peak of activity was significantly advanced in *Pdf* G4 x miR-277 SP line compared to two of the controls, but not with respect to the UAS miR-277 SP control (Kruskal Wallis, Dunn’s *post hoc* multiple comparisons. *Pdf* G4 x Scrambled SP vs. *Pdf* G4 x miR-277 SP *p* < 0.001, *Pdf* G4 Control vs. *Pdf* G4 x miR-277 SP *p* < 0.001, UAS miR-277 SP control vs. Pdf G4 x miR-277 SP *p <* 0.99). Under 100 lux, the experimental line showed a significant advance in phase of the M peak compared to the three controls (Kruskal Wallis, Dunn’s *post hoc* multiple comparisons. *Pdf* G4 x Scrambled SP vs. *Pdf* G4 x miR-277 SP *p* < 0.01, *Pdf* G4 Control vs. *Pdf* G4 x miR-277 SP *p* < 0.001, UAS miR-277 SP control vs. *Pdf* G4 x miR-277 SP *p* < 0.01). **(F)** Under 1000 lux light intensity, the phase of morning peak of *Pdf* G4 x miR-277 SP line does not exhibit any significant difference compared to that of the controls (Kruskal Wallis, Dunn’s *post hoc* multiple comparisons. *Pdf* G4 x Do. Scr. Sponge vs. *Pdf* G4 x miR-277 Sponge *p* = 0.99, *Pdf* G4 Control vs. *Pdf* G4 x miR-277 Sponge *p* = 0.99, UAS miR-277 SP control vs. *Pdf* G4 x miR-277 SP *p* = 0.99).

Since the activity around lights-on is altered when miR-277 was downregulated in the PDF neurons, we decided to check how different light intensities might impact the activity-rest rhythm. To this end, we downregulated miR-277 in the PDF neurons and recorded their activity rest rhythm under LD with three different light intensities *viz* 10 lux, 100 lux and 1000 lux. Under 10 lux, we observed that the experimental flies failed to have a proper M peak of activity (Figure 3A). Instead, they showed a sustained increase in activity starting nearly 2 hours before lights-on (ZT22.25–23.05). Besides, these flies exhibited a significant reduction in the activity immediately after lights-on and the evening activity onset was delayed (Supplementary Figure S1H). However, the phase of the M peak was not significantly advanced with respect to the UAS miR-277 Sponge control (Figure 3B). Under 100 lux, miR-277 downregulated flies exhibited significant increase in activity at some time points before lights-on (ZT22.75 and 23) (Figure 3C). Upon phase calculation, the M peak was found to be significantly advanced with respect to the controls (Figure 3D). In addition to the altered activity around the M peak, the experimental flies exhibited a significantly decreased activity before lights-off and increased activity after the lights-off under both 10 lux and 100 lux. But no significant difference was found in E activity onset under 100 lux (Figure 3A, C, Supplementary Figure S1I). When we further increased the light intensity to 1000 lux, experimental flies showed a significant increase in activity at ZT 22.75 but decreased activity at ZT 0.50 compared to that of the control flies (Figure 3E). The miR-277 downregulated flies did not exhibit any significant difference in the phase of the morning peak under high light intensity compared to the controls (Figure 3F). The evening activity onset also was not significantly different from the controls (Supplementary Figure S1J). These results suggest that miR-277 present in the PDF neurons is critical for the proper activity distribution around the M peak at various light intensities as well as in the activity regulation around E peak under lower light intensities.

### Downregulation of miR-277 in the PDF neurons abolishes *Clk* mRNA oscillation

**FIGURE 4:**
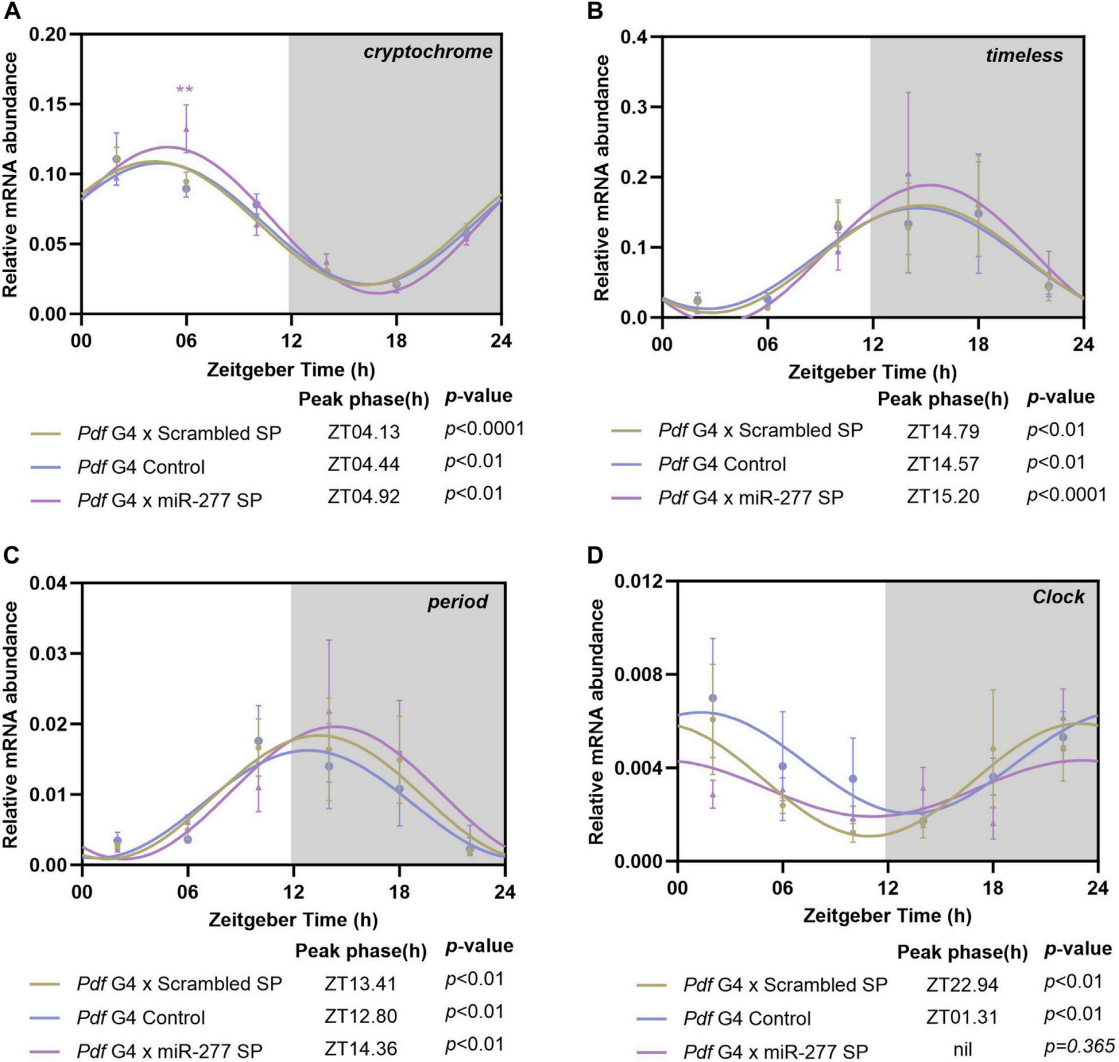
Transcript oscillation of various clock genes in the whole head extract of flies with miR-277 downregulation in the PDF neurons under LD. Transcripts were analysed at 6 different time points across the day with 4 h intervals. *y*-axis represents the transcript value of the clock genes normalised to the respective *rp49* value at each time point. Three biological replicates were used for each time point and mean value was plotted with the SEM as the error bar. The values at these time points were fitted into a sine wave to obtain the rhythm parameters such as acrophase, amplitude, *etc.*, *p*-value less than 0.05 indicates oscillation of the gene. Grey shaded region indicates the dark phase under LD. **(A)** *cryptochrome (cry)* transcript level exhibits significant oscillation with peak occurring between ZT04-ZT05 in the experimental as well as the controls (Cosinor analysis. *Pdf* G4 x Scrambled SP *p* < 0.001, *Pdf* G4 Control *p* < 0.01, *Pdf* G4 x miR-277 Sponge *p* < 0.01). The *cry* transcript level in the experimental fly heads at ZT06 was found to be significantly increased compared to the controls (Two way ANOVA, Tukey’s HSD test. *Pdf* G4 x Scrambled SP vs*. Pdf* G4 x miR-277 SP *p* < 0.01, *Pdf* G4 Control vs*. Pdf* G4 x miR-277 SP *p* < 0.001). **(B, C)** *timeless (tim)* and *period (per)* transcript oscillation in the *Pdf* G4 x miR-277 SP fly heads compared to *Pdf* G4 control and *Pdf* G4 x Scrambled SP control under LD. Both the transcripts exhibit significant cycling with peak between ZT12-15 in the experimental as well as in the control flies. (Cosinor analysis for *tim*. *Pdf* G4 x Scrambled SP *p* < 0.01, *Pdf* G4 Control *p* < 0.01, *Pdf* G4 x miR-277 SP *p* < 0.001. Cosinor analysis for *per. Pdf* G4 x Scrambled SP *p* < 0.01, *Pdf* G4 Control *p* < 0.01, *Pdf* G4 x miR-277 Sponge *p* < 0.01). **(D)** Control flies exhibited significant oscillation of *Clk* mRNA with peak between ZT23-ZT02. However, *Clk* mRNA diurnal oscillation was abolished in the fly head when miR-277 was downregulated in the PDF expressing clock neurons (Cosinor analysis. *Pdf* G4 x Scrambled SP *p* < 0.01, *Pdf* G4 Control *p* < 0.01, *Pdf* G4 x miR-277 SP *p* = 0.365).

In order to test whether the effect of miR-277 on the phasing of the morning peak is mediated by CRY, we downregulated miR-277 in the PDF neurons and the mRNA level of *cry* was analysed at 6 different time points across the day with 4h interval. The *cry* was found to oscillate in the controls as well as in the experimental fly heads with a peak around ZT04 (Figure 4A). The mRNA level of *cry* at ZT06 was found to be significantly higher in miR-277 downregulated flies compared to the controls (Figure 4A). This might be suggestive of the role of miR-277 in regulating the *cry* transcript levels in *Drosophila* under LD.

To understand the effect of downregulation of miR-277 in the PDF neurons on the core clock machinery, the mRNA oscillations of *tim* and *per* were analysed. Both *tim* and *per* mRNAs showed oscillations when miR-277 was downregulated in the PDF neurons (Figures 4B, C), with the peak of the occurring between ZT12-15 in the experimental as well as the control fly heads. We further analysed the transcript oscillation of *Clk* and it was found that both Gal4 and scrambled sponge controls exhibit oscillations with a peak close to lights-on (Supplementary Figure S1K). However, to our surprise, we found that the *Clk* mRNA oscillation is abolished in the fly head when miR-277 is downregulated in the PDF neurons (Figure 4D). Thus, we find that presence of miR-277 is critical for the maintenance of oscillating *Clk* mRNA levels. However, the abolishment of the *Clk* oscillation was not found to affect the *tim* and *per* oscillation.

## Discussion

Although miRNAs are important regulators of diverse physiological processes, our understanding of their potential roles in maintaining proper clock function is still limited. Recent studies showed that miRNAs are involved in maintaining the phase, amplitude and period of the circadian clock (Kadener et al., 2009; Mehta and Cheng, 2013; Chen et al., 2014; Garaulet et al., 2016; Xue and Zhang, 2018). In the present study, we assessed the role of miR-277 in regulating the circadian time keeping in *Drosophila*. In order to investigate the role of miR-277 in the clock tissues including the PDF positive clock neurons, miR-277 was downregulated using *tim* G4 and *Pdf* G4. Under LD, downregulation of miR-277 in the entire clock neuronal circuit under *tim* G4 resulted in an increased activity prior to lights-on at ZT23. These flies also exhibited some increased activity after lights-off. Under DD, the robustness of the rhythm was found to be reduced. Downregulation of miR-277 in the PDF-positive neurons, the principal regulators of the M peak, resulted in the advancement of the morning peak phase by about 40 min. The amplitude of the rhythm was reduced in the experimental flies indicating the importance of miR-277 in maintaining robust rhythms under DD. However, we did not find any significant difference in the free-running period of these flies indicating that the phase advancement observed in the experimental flies is not due to a change in the period of the circadian clock. miR-124 KO flies are known to have a similar effect, where the circadian phase is advanced without any change in the circadian period (Zhang et al., 2016). miR-210 KO flies also have an advancement in the phase of the evening peak while having normal FRP, amplitude and PER cyclic expression (Cusumano et al., 2018). Previous studies have shown that at higher temperatures, DN1p neurons promote the morning anticipatory activity without affecting the free-running period because it is temperature-compensated (Pittendrigh, 1954; Lamaze et al., 2017). Similarly, *glass* ^*60j*^ mutants which do not have DN1p are rhythmic under DD but do not exhibit morning anticipatory activity (Helfrich-Förster et al., 2001). The results of our study suggest that the M peak phase advancement observed in flies upon miR-277 downregulation in the PDF neurons is without affecting the speed of the clock. The amplitude of DD rhythm was found to be decreased in the clock/ PDF neurons specific downregulation of miR-277 indicating the importance of miR-277 expression in clock neurons for robust rhythms.

The role of miR-277 in the clock neurons was further investigated by overexpressing miR-277 in the whole clock tissues. This also resulted in a phase advance of the morning peak by a magnitude of approximately 40 min when compared to the controls. In addition, these flies enhanced the activity prior to lights-off. However, when miR-277 was overexpressed in PDF neurons, we found a phase advance in the morning peak of activity without an effect on the activity prior to lights-off. The FRP however was not different for the miR-277 overexpressed flies compared to the controls. This indicates that the overexpression of miR-277 in the PDF neurons might be affecting the PDF signaling from sLNvs to DN1ps and hence altering the activity around M peak without changing the speed of the clock (Zhang et al., 2010). Downregulation as well as upregulation of miR-277 in PDF neurons specific manner producing a similar behavioural phenotype in the phase of morning activity peak is perplexing. Several studies have previously reported similar instances where miRNA downregulation as well as overexpression showing similar phenotype. Downregulation as well as overexpression of miR-276a in the clock neurons is reported to result in decreased rhythmicity (Chen and Rosbash, 2016). Constitutive expression as well as downregulation of miR-277 is known to result in reduced lifespan in flies (Esslinger et al., 2013). This study identified miR-277 as a regulator of branched chain amino acid (BCAA) catabolism and proposed that fine tuning of BCAA catabolism by miR-277 is required for optimal longevity. MicroRNAs are known to fine tune the expression of target genes to physiologically relevant levels (Hobert, 2007; Karres et al., 2007). Hence, we assume that the level of mRNA responsible for phasing of M peak might be fine-tuned to an optimal level by miR-277 and downregulation or overexpression of miR-277 is possibly altering the optimal expression of the target mRNA, leading to phase advancement.

Previous reports suggest that artificially overexpressing miRNAs may cause non-physiological effects (Selbach et al., 2008). Some studies discuss the possibility that overexpression of miRNA could cause downregulation of physiologically non relevant target mRNAs or mRNAs that have weaker affinity binding sites (Schertel et al., 2012). Hence it is possible that the functional target might be different during downregulation and overexpression of the miRNA as the miRNA-target interaction dynamics is dependent on their relative expression levels. Hence the phase advancement observed in downregulation and overexpression of miR-277 might be mediated by two distinct target mRNAs acting on the same or different pathway of M peak phase regulation in the PDF neurons. We also observe that robustness of the rhythm is reduced upon downregulation of miR-277 in the clock neurons but not upon overexpression. This also might be indicative of the fact that two distinct mRNAs are being targeted during downregulation and overexpression.

Since light is the only zeitgeber in an experiment set up with constant temperature and humidity settings, the altered phase under LD condition can be assumed to be the result of changes in the light sensory pathways or due to changes in the core clock components. Blue light photoreceptor CRY and other external photoreceptors are thought to be important in light mediated entrainment (Stanewsky et al., 1998; Helfrich-Förster et al., 2002; Rieger et al., 2003). We assessed the role of miR-277 in governing the phase of behavioural rhythm under various light intensities. Downregulation of miR-277 in PDF-positive neurons altered the activity around the morning peak under LD with various light intensities ranging from 10 to 1000 lux indicating that miR-277 expressed in PDF-positive neurons mediate the proper activity distribution around the morning peak under various light intensities. Whereas we found a difference in the activity around the E peak only at lower light intensities. This might be because the morning oscillator is capable of interacting with the evening oscillator via the PDF neuropeptide and modulates the timing of the evening behaviour (Cusumano et al., 2009). Eventhough both LNds as well as DN1ps are capable of setting the phase of the evening activity, under low light intensities, the morning oscillator enslaves the LNd evening oscillator and this interaction diminishes with increasing light intensity (Chatterjee et al., 2018). miR-277 downregulation in the PDF neuron affected the activity around the evening peak only at lower light intensity and this effect is diminished under high light intensity (1000 lux). Hence, we speculate that miR-277 present in the PDF neurons might be influencing the phasing of the rhythm by controlling the LNv-LNd interaction, possibly via PDF signalling.

The difference in locomotor activity response during light-dark or dark-light transitions under various light intensities of LD might be due to the difference in the entrainment pathways under different light intensities. *norpA* encoding Phospholipase-C (PLC), which acts downstream of rhodopsin (RH) is critical for entrainment under low light intensities (Zuker, 1996). CRY and 4 Rhodopsin expressing photoreceptors (R1-R6 expressing RH1, yellow R8 inner photoreceptor expressing RH6, pale R7 photoreceptor expressing RH3 and yellow R7 photoreceptor expressing RH4) of compound eyes are also known to be involved in entrainment at low intensities (Stanewsky et al., 1998; Saint-Charles et al., 2016). Entrainment at higher intensities is known to be mediated by HB eyelets possibly with the help of RH6 in a *norpA*-independent pathway (Schlichting et al., 2019).

Misregulation of miR-277 in the PDF positive neurons did not alter the phase of *cry* transcript diurnal oscillation, however we found a significant increase in the *cry* mRNA level at ZT06 in these flies compared to the controls. We further analysed the impact of miR-277 expressed in PDF positive neurons on the central oscillator core clock genes such as *per*, *tim* and *Clk*. When miR-277 was downregulated in PDF positive neurons, we did not find any significant difference in the *tim* and *per* transcript oscillation. Nonetheless, downregulation of miR-277 in the PDF neurons abolished *Clk* mRNA oscillation in the fly head. Such an effect on the *Clk* oscillation upon downregulation of the miRNA in a very small subset of clock neurons suggests the possibility of miR-277 expressed in the PDF neurons affecting the clock machinery in the visual photoreceptors. Clock as well as clock regulated genes are expressed in the different cell types of *Drosophila* visual system and circadian neuropeptides such as PDF and Ion transport peptide (ITP) are shown to regulate circadian rhythms in the expression of these genes (Damulewicz et al., 2013; Górska-Andrzejak et al., 2013; Damulewicz et al., 2015). Large LNvs have arborizations extending to the distal medulla and PDF receptors are expressed in the fenestrated glia of the outer retina, suggesting potential PDF signalling to the photoreceptor cells (Im and Taghert, 2010).

When we downregulated miR-277 in PDF positive neurons, *Clk* mRNA oscillation was abolished but it didn’t affect the *per* and *tim* oscillation. Although the *Clk* mRNA exhibits circadian oscillation in wild type flies, the CLK protein level is not known to exhibit any daily oscillation (Bae et al., 1998; Houl et al., 2006). The *Clk*ARK flies with *Clk* mRNA expression under the control of *per* or *tim* promotor exhibited normal *per* and *tim* oscillations and had no effects on the circadian regulation of locomotor activity of the flies except the extended morning activity (Kim et al., 2002). In addition, simulation studies showed that fixing the concentration of the CLK to a constant level or to half of its peak value does not affect the circadian oscillation of *per* or PER (Smolen et al., 2001; Smolen et al., 2004). Taken together these studies support the observation that *Clk* mRNA oscillation can be abolished without necessarily impacting the *per* or *tim* oscillations.

In order to identify whether *Clk* is under post-transcriptional regulation of miR-277, we looked at data obtained from previous *in vivo* study of miRNA regulated transcripts (Xia et al., 2020). The CLEAR-CLIP data does not reveal any interaction between *Clk* and miR-277. This was further confirmed by *in silico* analyses. These bioinformatic tools uses various criteria such as complementarity between the seed region of the miRNA and seed match region in the 3′UTR of the mRNA, the free energy change of the hybridisation, evolutionary conservation of the miRNA-mRNA pair, *etc*., to predict the targets of a miRNA. We have used PicTar, TargetScanFly7.2 and Diana Tools for target prediction. *Clk* or any of the transcripts in the primary TTFL did not come up in the analyses using these softwares. Hence it is likely that miR-277 does not directly target the *Clk* and it regulates the *Clk* oscillation via a mediator. Since *Clk* gene expression is regulated by PDP1 and VRILLE (VRI) (Cyran et al., 2003), we also checked whether these mRNAs are targets of miR-277. Both CLEAR-CLIP and Diana Tools software suggest *pdp1ε* as a potential target, suggesting a possible mechanism by which miR-277 might be affecting *Clk* transcript oscillation.

Studies using the *Clk* ^*Jrk*^ mutants have shown that this mutation abolishes the lights-on locomotor activity response and these flies fail to anticipate light to dark transition under LD. *ClkSV40* flies with deleted 3′UTR site exhibit defects in the development of the PDF neurons with unequal number of PDF neurons in both the hemispheres (Lerner et al., 2015). These studies along with our results point to the importance of miR-277 mediated proper *Clk* expression in the PDF neurons which in turn may govern the clock behavioral output. Although *Clk* mRNA level was measured in the adult fly head, the *Pdf* G4 driven miR-277 downregulation was not adult stage specific. Hence we cannot tease apart the developmental and adult stage specific roles of miR-277 in the PDF neurons.

In summary, our study shows a previously unknown role of miR-277 in governing the phase of circadian locomotor activity rest rhythm in *Drosophila*. The results of our study suggest that miR-277 expressed in the PDF neurons controls circadian rhythms in flies probably by fine tuning the *Clk* diurnal oscillations.

## Data Availability

The original contributions presented in the study are included in the article/Supplementary Material, further inquiries can be directed to the corresponding author.
