# Supplementary material for: miR-277 regulates the phase of circadian activity-rest rhythm in Drosophila melanogaster
Source: Front Physiol. 2023 Nov 28;14:1082866. doi: 10.3389/fphys.2023.1082866 (PMC10714010; doi:10.3389/fphys.2023.1082866)
Supplement: Supplementary file 1 [file Table1.pdf]

Table S1: List of primers used

Gene	**Forward primer 5’-3’**	**Reverse primer 5’-3’**
rp49	GCTAAGCTGTCGCACAAA	TCCGGTGGGCAGCATGTG
cry	GCAGTACGTCCCGGAGTTGA	AGGGCTCGTGAACAAATTCCT
per	GAGCTGAGTGACATATAGCC	TTGACTAGTGCGAGATTACAC
tim	CCGTGGACGTGATGTACCGCAC	CGCAATGGGCATGCGTCTCTG
Clk	GGATAAGTCCACGGTCCTGA	CTCCAGCATGAGGTGAGTGT
