# Supplementary figures and images for: miR-277 regulates the phase of circadian activity-rest rhythm in Drosophila melanogaster
Source: Front Physiol. 2023 Nov 28;14:1082866. doi: 10.3389/fphys.2023.1082866 (PMC10714010; doi:10.3389/fphys.2023.1082866)

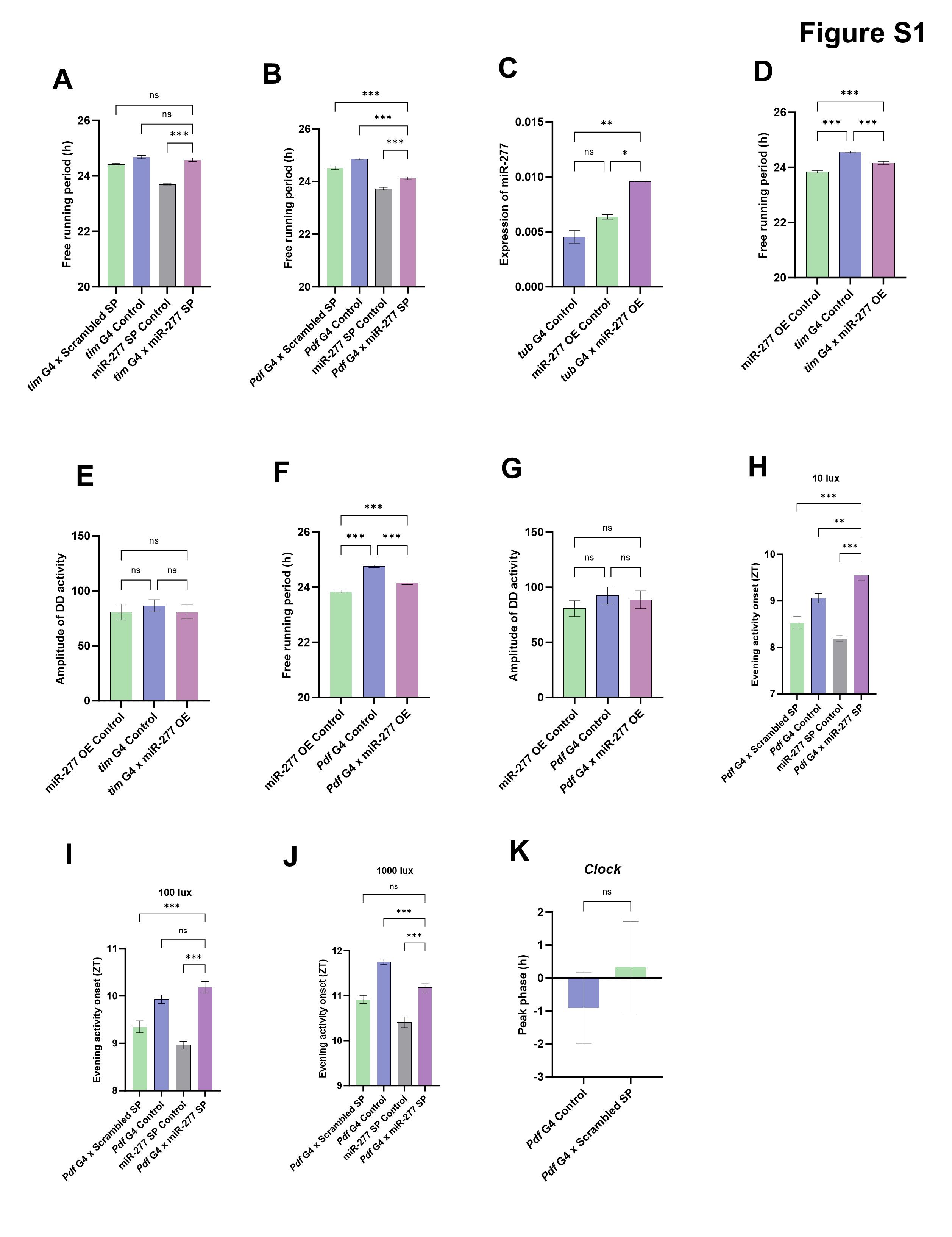

Supplement: Supplementary file 3 [file Image1.jpg]
